# Conceptual development of “at-homeness” despite illness and disease: A review

**DOI:** 10.3402/qhw.v9.23677

**Published:** 2014-05-26

**Authors:** Joakim Öhlén, Inger Ekman, Karin Zingmark, Ingrid Bolmsjö, Eva Benzein

**Affiliations:** 1 Palliative Research Centre, Ersta Sköndal University College and Ersta Hospital, Stockholm, Sweden; 2 Institute of Health and Care Sciences, The Sahlgrenska Academy, University of Gothenburg, Sweden; 3 University of Gothenburg Centre for Person-Centred Care, Gothenburg, Sweden; 4 Research and Development Unit, Norrbotten County Council, Luleå, Sweden; 5 Department of Health Sciences, Luleå University of Technology, Luleå, Sweden; 6 Department of Care Science, Malmö University, Malmö, Sweden; 7 Department of Health and Caring Sciences, Linnæus University, Kalmar, Sweden; 8 Center for Collaborative Palliative Care, Linnæus University, Kalmar, Sweden

**Keywords:** At-homeness, concept development, home, literature review, wellness, well-being

## Abstract

Only one empirical study, the one by Zingmark, Norberg and Sandman published in 1995, explicitly focuses on at-homeness, the feeling of being metaphorically at-home, as a particular aspect of wellness. However, other studies reveal aspects of at-homeness, but if or how such aspects of at-homeness are related to each other is unclear. For this reason, the aim was to review Scandinavian nursing research related to at-homeness in the context of wellness–illness in severe and long-term conditions in order to take a step towards conceptual clarification of “at-homeness.” The review included interpretive studies related to severe and long-term illness conducted in Sweden: 10 original articles and 5 doctoral theses. “At-homeness” was found to be a contextually related meaning of wellness despite illness and disease embedded in the continuum of being metaphorically at-home and metaphorically homeless. This was characterized by three interrelated aspects and four processes: being safe through expanding–limiting experiences of illness and time, being connected through reunifying–detaching ways of relating, and being centred through recognition–non-recognition of oneself in the experience and others giving–withdrawing a place for oneself. This conceptualization is to be regarded as a step in conceptual clarification. Further empirical investigation and theoretical development of “at-homeness” are needed. The conceptualization will be a step of plausible significance for the evaluation of interventions aimed at enhancing wellness for people with severe long-term illness, such as the frail elderly, and people with chronic illness or palliative care needs.

The experience of at-homeness in Scandinavian nursing research has been described as a feeling of being metaphorically at-home in spite of illness and disease. The emphasis in this experience is on existential dimensions, where suffering and distress threaten feelings of being at-home (Ekman, Skott, & Norberg, 2001; Rasmussen, Jansson, & Norberg, 2000; Zingmark, Norberg, & Sandman, 1993). Other aspects of at-homeness have been shown to be related to the meaning of hope (Benzein, 1999), living with severe chronic illness (Ekman, 1999) and alleviation of suffering at the end-of-life (Öhlén, 2000). Other studies present similar results (Edvardsson, Rasmussen, & Riessman, 2003; Talseth, Gilje, & Norberg, 2003). However, it is not known if or how such aspects of at-homeness are related to each other.

Zingmark, Norberg, and Sandman (1995) suggest several common interdependent and interrelated aspects of at-homeness including the sense of being related to oneself, significant others, things, places and activities, and transcending time and space. These authors described integral parts of at-homeness throughout the life span as “being given a home,” “creating a home,” “sharing a home” and “offering a home.” A preliminary conceptual development of environmental influences in palliative care by Rasmussen and Edvardsson (2007) suggests at-homeness as an experiential outcome of atmospheres of hospitality, safety and everydayness. Their research was based on four empirical studies conducted by their team and did not elaborate on the concept of at-homeness. A similar conceptual development in terms of “homelikeness” as an aspect of existential well-being as opposed to “unhomelikeness” and “uncanniness” as essential experiences of illness, derived from phenomenological philosophy, has been put forward by Svenaeus (2000a, b).

The related literature on at-homeness outside of the Scandinavian arena is sparse. We found illness-related international studies with similar focus on the metaphoric meaning of home (Angus, Kontos, Dyck, McKeever, & Poland, 2005; De Veer & Kerkstra, 2001; Dyck, Kontos, Angus, & McKeever, 2005; Hammer, 1999; Moloney, 1997; Roush & Cox, 2000; Swenson, 1998) and the opposite: the metaphoric meaning of being homeless (Baumann, 1993) as well as conceptual clarifications thereof (Carboni, 1990). Molony, McDonald, and Palmisano-Mills (2007) developed the Experience of Home Scale, a paper and pencil questionnaire measuring existential place and particularly the strength of the experience of person–environment meaningfulness. Later, Molony (2010) synthesized home metaphors from 23 interpretive studies pertaining to the meaning of home or adjustment to home for people aged 65 and over as a physical and existential empowering refuge for relationship, personal reconciliation and integrating transitional processes promoting a sense of self. Other studies focus instead on meanings related to multidimensional environmental aspects of at-homeness: actually being at home or beyond the house (Cloutier-Fisher & Harvey, 2009) or a literal as well as metaphorical sense of welcome spatial dwelling (Galvin & Todres, 2011). Of the international studies, the ones by Molony (2010) and Molony et al. (2007) refer to aspects of at-homeness similar to Zingmark et al. (1995).

The suggestion that “at-homeness” is of significance for people with severe illness is in line with research emphasizing the communicative and contextual dynamics of wellness (Edvardsson, Sandman, & Rasmussen, 2006; Thorne et al., 2005). According to Jensen and Allen (1994), wellness–illness is the experience of health–disease; a struggle to “find a place where one is fulfilled and connected to the surrounding world” (p. 362), influenced by inter-, intra- and extra-personal dimensions. In wellness, we belong to various groups and have a sense of belonging, while illness means a loss of identity and a re-evaluation of what is meaningful in life. In this way, sensitivity is needed in helping vulnerable, ill people to maintain their wellness, which among other things involves being at home (Ekman et al., 2001; Rasmussen et al., 2000; Zingmark et al., 1993) and being known (Thorne et al., 2005) where this fragility exposes the person to the risk of having their dignity violated (Öhlén, 2004). Hence, wellness means being in a place or situation that encompasses a body integrated with the environment and the experience of illness characterized as a limited relationship between body, self and environment (Jensen & Allen, 1994). Consequently, the spatiality aspect of wellness or “at-homeness” needs further elucidation.

To seek conceptual clarity, and include the prospect of outcome measures of interventions aimed at enhancing wellness and at-homeness, we set up a project that interpretatively sought to integrate results related to at-homeness despite illness manifestations. We started this with a tentative definition, that is, the experience of metaphorically being at-home as described above by Zingmark et al. (1995). However, while research with direct focus on at-homeness was scarce, we found a variety of studies which highlighted the phenomenon, and consequently, the studies at hand seems to describe different aspects thereof. Assuming the potential of synergy effects, we conducted a review directed at concept clarification on research related to “at-homeness.” Consequently, the aim was to review Scandinavian nursing research related to at-homeness in the context of wellness–illness in severe and long-term conditions in order to take a step towards conceptual clarification of “at-homeness.”

## Method

The literature search for this review was an iterative process whereby eligible studies were found stepwise through a combination of search strategies (Conn et al., 2003). Because “at-homeness” is not a word used in computerized databases, we first explored the possibility of open literature searches in databases with keywords including “at-homeness,” “at-home,” “home” and “place.” Because a considerable number of items were found using “home” and “place” as keywords, we tried different search combinations in order to find studies related to metaphorical meanings of at-homeness. We found few such publications. We then conducted a renewed systematic literature search in the databases Cumulated Index of Nursing and Allied Health, PubMed, Scopus and PsychINFO covering three decades up until 2012 with the keywords “at-homeness,” “at-home,” “being at home,” “feeling at home,” “homecoming,” “coming home” “homeless,” “being homeless,” “homelessness,” “feeling homeless,” “home,” “place,” “placeless,” “placelessness,” “alienation” and “alien,” and forward and backward citation searches of studies already known to us or retrieved from the databases, particularly from the oldest and most informative studies. In total, this resulted in 338 publications, of which 167 were doubles, 102 investigations of study contexts other than disease and care related and 59 based on data outside Scandinvia. In total, this resulted in 10 articles. The ultimate inclusion criteria were results pertaining to severe and long-term illness, explicitly stated as displaying metaphorical and experiential aspects related to at-homeness from a patient/client perspective, conducted in Scandinavia and published in English or Swedish. The exclusion criteria were illness primarily related to stress disorders, psychiatric disorders apart from dementia, maternity and paediatric care, and home as a demarcated physical place.

Among these items, we recognized studies arising from Swedish theses, and in such cases we reviewed these, which resulted in the inclusion of five theses providing additional results to the articles.

Study contexts were Swedish palliative and end-of-life care home and hospice settings, residential care for elderly and group dwelling for people with dementia. Most studies were based on interview data, while two studies were based on observations; in total, there were 132 participants. The majority of the studies used phenomenological, hermeneutical (including lifeworld phenomenological) analytic approaches, one qualitative content analysis and two constant comparative analyses (see Table I).

**Table I:** Aim, context, sample, field method and data analysis: characteristics of the studies included in the review.

Author	Study aim	Context	Sample	Field method	Data analysis
Benzein (1999); Benzein, Norberg, & Saveman (2001)	To explore the meaning of the lived experience of hope in dying patients	Palliative home care	4 women and 7 men aged 54–83	Narrative interviews	Phenomenological hermeneutic
Ekman (1999)	To highlight aspects of the life situation of elderly people living with moderate to severe chronic heart failure (CHF)	Elderly with chronic heart failure CHF in Sweden	16 women and men aged 75–94	Narrative interviews	Phenomenological hermeneutic
Ekman, Skott, & Norberg (2001)	To achieve a deeper understanding of the meaning of the lived experience of being an elderly woman with CHF	Elderly with chronic heart failure CHF in Sweden	1 woman aged 76	2 interviews 1 year apart	Phenomenological hermeneutic
Elofsson & Öhlén (2004)	To gain deeper understanding of the meaning of the lived experiences of severely ill elderly people who have obstructive pulmonary disease and are in need of everyday care	Elderly with chronic obstructive pulmonary disease	2 women and 4 men aged 78–88	Dialogue interviews	Phenomenological hermeneutic
Erikson, Park, & Tham (2011)	To examine the shifting relationship between meaning, place, and activities during the year-long rehabilitation process	Rehabilitation for people with stroke	4 men and 3 women aged 42–61	Longitudinal interviews; four times	Constant comparative method
Heikkila & Ekman (2003)	Where do the elderly Finnish immigrants in Sweden want and expect to be cared for?	Elderly Finnish immigrants in Sweden	4 men and 35 women aged 75–89	Theme guided interviews	Latent qualitative content analysis
	What aspects affect wishes and expectations of care?				
Graneheim (2004); Graneheim & Jansson (2006)	To illuminate the meaning of living with dementia and disturbing behaviour, as narrated by three persons admitted to a residential home.	A residential home for people with dementia and complications that mainly take the form of disturbing behaviour	1 women and 2 men aged 73–79	Repeated informal conversational interviews	Phenomenological hermeneutic
					
Lindahl, Sandman, & Rasmussen (2003)	To highlight the meanings of being dependent on a ventilator and living at home	Swedish home care	6 women and 3 men	Interviews	Phenomenological hermeneutic
Öhlén (2000); Öhlén, Bengtsson, Skott, & Segesten (2002)	To explore meanings of alleviated suffering in people living with life-threatening cancer	Inpatient hospice and palliative home care	16 women and men aged 53–88	Repeated conversations	Lifeworld phenomenological
Rasmussen, Jansson & Norberg (2000)	To show the effects of nursing care as experienced by hospice patients	Hospice care	2 men and 10 women aged 32–95	Conversationalresearch interviews	Phenomenological hermeneutic
Zingmark, Norberg & Sandman (1993)	To gain deeper understanding of demented patients’ everyday life and explore demented patients’ behaviour related to home	A group dwelling for dementia patients	6 women aged 65–79	Participant and non-participant observations	Constant comparative method
Zingmark (2000)	To highlight the meaning of home-related experiences in people with advanced Alzheimer disease living in a homelike care setting, and moments of homecoming disclosed in a woman with Alzheimer disease in an advanced stage who mostly talked about going home and being lost*	A group dwelling for demented patients	6 women	Participant observations of care episodes over 20 months	Phenomenological hermeneutic and case study

* We refer to studies II and III of this thesis.

A review approach with emphasis on clarifying conceptualizations regarding the phenomenon of at-homeness was chosen. We found that all the articles selected for review were performed by means of interpretative analysis, and this led us to choose to use such an approach for our review. First, a reading of the texts as a whole aimed at formulating interpretative questions to be verified in the following analysis: Which aspects, processes and dimensions of at-homeness can be discerned? Can relationships thereof be stated? If so, which? These questions were then used to reveal patterns of meaning structures. To achieve this, isolated results in each included study that were especially descriptive of the phenomenon were focused, including study contexts and themes (Jensen & Allen, 1994). Each of the studies was analysed in detail by one of the authors and then discussed among all the authors, searching for diverse structures displaying significant aspects of the phenomenon. Finally, we sought a common structure within which the reviewed studies could be translated into each other. In doing this, aspects of the phenomenon were sorted in terms of similarities and differences by interchangeably reading the texts and having reflective discussions in the group, thus gradually revealing aspects and processes of at-homeness. For the presentation below, we start with a conceptual overview of at-homeness and then clarify meaning structures: aspects, dimensions and processes.

## Findings

**Figure 1:**
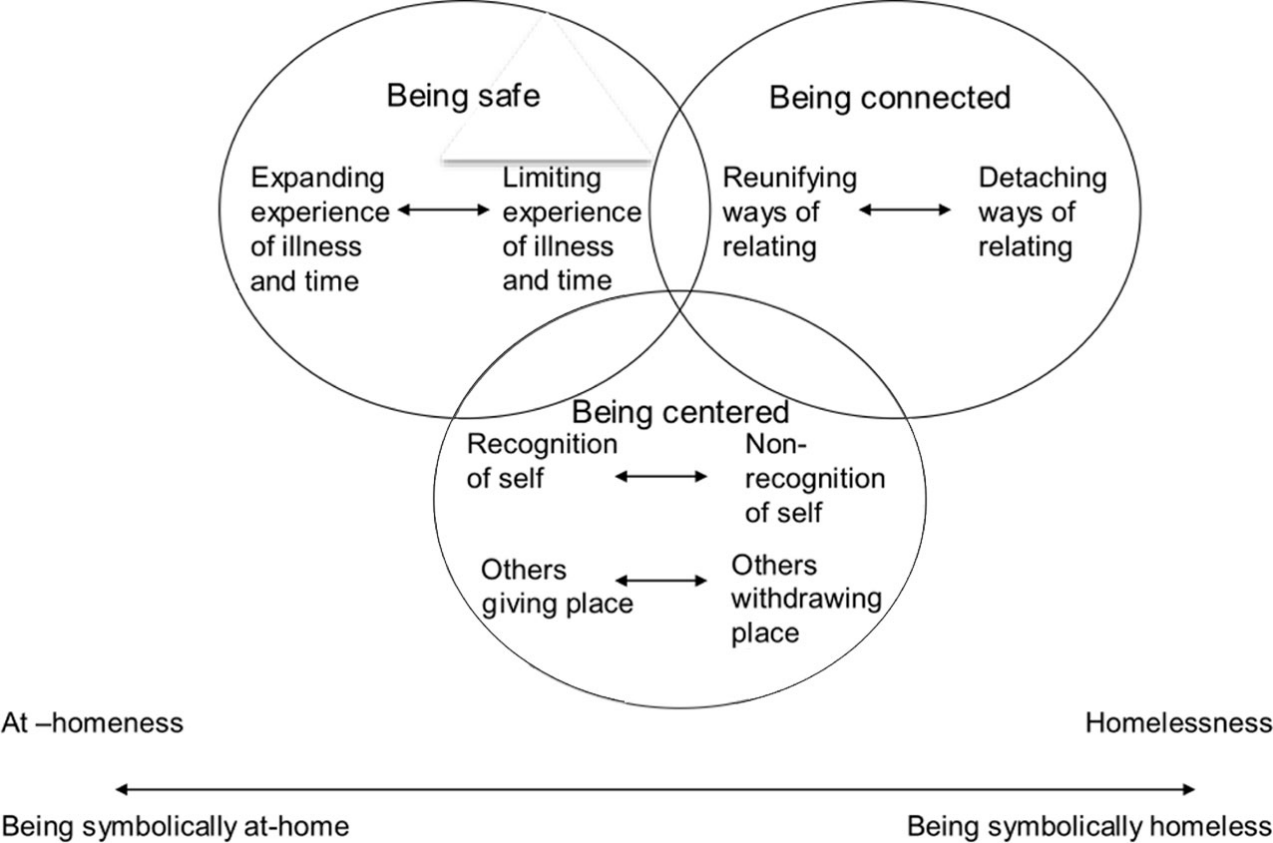
Aspects of being at-home as related to four processes in the at-homeness–homelessness continuum dimension.

Of the 10 articles and 5 theses reviewed (for overview of the studies see Table I), at-homeness despite illness and disease was interpreted as containing three interrelated aspects: (i) being safe, (ii) being connected and (iii) being centred. In addition, at-homeness presents itself on a continuum dimension from being metaphorically at-home to its opposite, being metaphorically homeless. From this, four processes, which both enhanced and hampered at-homeness, were involved in this continuum: (a) at-homeness as being safe through expanding–limiting experiences of illness and time, (b) at-homeness as being connected through reunifying–detaching ways of relating, (c) at-homeness as being centred through recognition–non-recognition of oneself in the experience and (d) others giving–withdrawing a place for oneself (see Figure 1). Each of these processes appears bi-directional: either towards the at-homeness pole or the homelessness pole.

As the above-mentioned three aspects of at-homeness are interrelated, each of them can be identified in a clinical situation, as illustrated by the following example from the study by Öhlén et al. (2002). The example is used as a paradigm example throughout the “Findings” section. An elderly woman who was severely ill with cancer described an ordinary day at the hospice where she was cared for:

Things are bad in the morning. I'm tired as hell. You have to lie there and think about how the day's going to be. I'm afraid of stress. I always get tea then and I usually sit on the couch there and drink it. And then I have milk and two pieces of toast. And in there [points towards the refrigerator in her room at the hospice] I have real butter, and marmalade, I think it's English, which I usually have. Yes, I have that everyday. I sit and enjoy myself for about an hour. That's my golden hour. Sometimes I take a shower first, but I don't always have the strength. Every morning is different. After my tea time I gradually wake up. By then I've gotten my medicine too. Dolcontin^®^ is important you know. Then around eleven I start moving around (Öhlén et al., 2002, p. 321).

In this narrative, there is an obvious paradox in that the mornings are associated with both the worst periods of the woman's day and her “golden hour.” Having the opportunity to do what she considered to be of importance for herself at her own pace made a significant difference to her. Significant things from her everyday life, for instance, the butter and English marmalade, signified her personal habits. Because of this, she could keep symptoms and distress at a distance and was able to relate to time and space in connecting ways, thus feeling safe despite severe illness manifestations due to advanced cancer, and also being centred in ways which gave her room for inner reflection. In this way, the “golden hour” could give the woman space to be centred and to reunify with the memory of people and events from her past. She also gave examples of when this was enhanced by the respectful actions of caregivers or not, such as when her breakfast tray was disrespectfully taken away (Öhlén, 2000). These contradictory statements about how her mornings included both bad moments and “golden hours” might illustrate the fragility of the experience and the two poles of the processes which enhance and hamper at-homeness: being at-home and being homeless despite illness.

### At-homeness as being safe

Being safe as an aspect of at-homeness despite illness is characterized by being free (Ekman, 1999) and independent (Öhlén, 2000) as well as being released from illness manifestations (Ekman, 1999; Rasmussen et al., 2000), suffering (Öhlén, 2000), burden and demands (Graneheim, 2004; Zingmark, 2000). The focus is on the present (Rasmussen et al., 2000; Zingmark et al., 1993; Öhlén, 2000), and even the moment (Rasmussen et al., 2000), with the opportunity to metaphorically transcend time and space (Benzein, 1999; Öhlén, 2000). A person can live a decaying body and have severe illness, but still feel safe by arousing a zest for life (Öhlén et al., 2002) and being aware of their possibilities (Benzein, 1999). This is characterized by a presentation of self which is separate from the “private” self, which might be connected to an ill and decaying body. In addition to this, the ill person can feel safe when the technology needed for support is integrated into the own home and a warm atmosphere at the ward can also be helpful in order to gain a feeling of security (Elofsson & Öhlén, 2004; Erikson et al., 2011; Lindahl et al., 2003).

The process that facilitates or hampers being safe is described as an expanding-limiting experience of illness and time, and related to the polar extremes of at-homeness and homelessness. In the paradigm example above, this is illustrated in the way the woman describes significant prerequisites for keeping the distress of her decaying body at bay. By taking her own time to reflect on how the day was going to be, she could regain and rediscover meaning in an otherwise limiting situation, thus enhancing the at-homeness experience itself. On the other hand, relief and alleviation from manifestations of illness, suffering, burden and demand facilitate at-homeness by limiting the homeless experience (Öhlén, 2000). At the core of this process are transcendence of disabilities, symptoms and health-related problems, which can be supported through integration of the place with wellness-enabling activities, such as the arrangements of aids in order to not challenge comfort zones in patient's homes (Elofsson & Öhlén, 2004; Erikson et al., 2011; Lindahl et al., 2003). In order to be safe in a limiting situation, at-homeness will also be enabled when you “rise above yourself and your personal boundaries” (Lindahl et al., 2003, p. 25).

### At-homeness as being connected

Being connected as an aspect of at-homeness despite illness is characterized by living in unifying companionships (Graneheim & Jansson, 2006; Zingmark, 2000) and being respectfully approached as a person (Öhlén, 2000; Rasmussen et al., 2000) in one's limiting existential situation (Öhlén, 2000) while experiencing an inner place of belonging (Ekman et al., 2001; Rasmussen et al., 2000). This is also described as being reached by language (Zingmark, 2000). Being connected can provide the critical link to finding meaning in life (Benzein, 1999). It occurs in a metaphorical place with others, and can be reached by the release of energy (Benzein, 1999), peaceful atmosphere (Zingmark et al., 1993) and trusting one's own judgment regarding decisions related to illness (Ekman, 1999). This can be facilitated in affirming relationships (Benzein, 1999; Graneheim & Jansson, 2006), particularly by respecting personal rhythms and having time both in the present and the past with oneself and with others (Zingmark, 2000; Zingmark et al., 1993). Contact with pets, the environment, nature and a higher being including destiny and God also contribute (Öhlén, 2000). Thus, being respectfully received by caregivers appears as an essential means to at-homeness (Ekman, 1999; Heikkila & Ekman, 2003).

The process of facilitating–hampering was described as being linked to reunifying–detaching ways of relating. As illustrated in the paradigm example, the woman stated that having time and space at the hospice gave her a metaphorical place of belonging and helped her reunite with herself and others. This type of time and space can facilitate and mediate at-homeness experiences, whilst the absence of such conditions may cause a person to relate in a detached way.

The polar extremes of at-homeness and homelessness were identified in the studies describing this process. Several studies describe reunifying ways of relating through respectful companionships (Benzein, 1999; Heikkila & Ekman, 2003; Öhlén, 2000; Rasmussen et al., 2000; Zingmark 2000; Zingmark et al., 1993). Rasmussen et al. (2000) describe detached ways of relating in disrespectful companionships, that is, how at-homeness can be hampered by fragmentation and alienation and is experienced as being displaced, unsafe and entrapped in silence. Detached ways of relating can result in feelings of homesickness and longing for a better place (Graneheim, 2004; Graneheim & Jansson, 2006).

### At-homeness as being centred

Lastly, the “being centred” aspect of at-homeness despite illness is characterized by experiencing a recognized self in an inner place of comfort, something that is particularly emphasized when a person lives a distressing and decaying body. Thus, at-homeness is a centre of self (Zingmark et al., 1993) and everyday experience, a metaphorical place or shelter where you can be ready to be who you want to be and a possibility for the “real” to be visible (Benzein, 1999; Graneheim, 2004). This includes being permitted a comfortable place for body and self, a secure (Lindahl et al., 2003), safe (Ekman, 1999) and familiar place (Benzein et al., 2001; Erikson et al., 2011), or a place which symbolizes the freedom experienced before severe long-term illnesses (Graneheim, 2004). These places are labelled “lived retreat” (Öhlén, 2000, Öhlén et al., 2002) and “sanctuary” (Rasmussen et al., 2000)—space for reflection on such things as the life, the self and the illness (Ekman et al., 2001). Experiencing such a place entails the existence of an inner space from which the person can reach out and is given ease (Lindahl et al., 2003).

Further, being centred is connected with a spirit of availability, congruent with one's outer and inner atmosphere and space. A special facet of being centred is the opportunity to transcend the actual moment by experiencing the presence of people, places or attributes from the past or future (Benzein et al., 2001; Öhlén, 2000). At-homeness includes an awareness of the body as being relaxed and peaceful (Rasmussen et al., 2000; Zingmark, 2000) in ways for the self to be recognized despite the illness. By being centred through experiencing the recognized self, the person can be and live in hope (Benzein et al., 2001).

Two processes appeared which facilitated–hampered being centred. First, recognition–non-recognition of oneself in the experience, which involves a predominantly intrapersonal process, and second, others giving–withdrawing a place for oneself—which conversely involves a predominantly interpersonal process. For both processes, the reviewed studies described both dimensions of being at-home and being homeless. In the paradigm example, this is illustrated in the significance the woman gave to her “golden hour” in which she describes being given a place to recognize herself. However, the opposite is also illustrated in cases when staff interrupted her centeredness and took her breakfast tray away (Öhlén, 2000). Further, being centred was found to release energy (Benzein, 1999) in a peaceful atmosphere (Zingmark et al., 1993). This appears to be an indication that the intra- and interpersonal processes are interrelated and might even be interconnected.

## Discussion

Conceptually, “at-homeness” despite illness and disease was found to be a contextually related meaning of wellness characterized by three interrelated aspects and four processes: being safe through expanding–limiting experiences of illness and time, being connected through reunifying–detaching ways of relating, and being centred through recognition–non-recognition of oneself in the experience and others giving–withdrawing a place for oneself. The experience of at-homeness is imbedded in the continuum of being metaphorically at-home and metaphorically homeless. Individuals are involved in an ongoing process to capture the perceived opportunity to establish an experience of at-homeness, that is, at-homeness may be seen as both a process and a state; and this result confirms Molony's (2010) study. Thus, at-homeness encompasses living and having a place where privacy, identity and safety can be preserved, protected (Roush & Cox, 2000; Williams, 2004) and enacted in the aesthetics of place and the changing potential and limitations due to illness (Angus et al., 2005; Dyck et al., 2005) and being respectfully seen as a person while experiencing an inner place of belonging (Edvardsson et al., 2003). Further, a sense of at-homeness can be regarded as an experiential focus of spatial possibilities (Galvin & Todres, 2011).

The conceptual clarification of at-homeness is in line with the description of the experience of wellness context, which, according to Jensen and Allen (1994) encompasses being a body taken for granted that in illness surfaces as a changing and mistrusting body, a struggling towards an uncertain future and a desire for being in relationships. Further, our analysis of at-homeness confirms the multi-relationality described in the interview study by Zingmark et al. (1995). They first described “relatedness” as a common condition of being at-home. The significance of meaningful relations with significant others, things, places and activities in order to achieve at-homeness was confirmed in our review. Meaningful relation with oneself or through transcendence was found in the aspect “being centred.” The situational and fleeting character of at-homeness, as it can appear on the continuum dimension of being metaphorically at-home as opposed to being metaphorically homeless, is underscored in the ways at-homeness is maintained throughout life, as described by Zingmark et al. (1995): being given a home, creating a home, sharing a home and offering a home. Further, the dynamics of at-homeness display interesting commonalities with Thorne et al.'s (2005) emerging conceptualization of “being or not being known” in healthcare communication. Their study discloses how various communicative patterns (in the context of cancer care) are needed by healthcare professionals in order to facilitate the human connection highly valued by patients, and highlights the shortcomings of standardized and routinized approaches to communication. Thus, how various contextual factors may shape the four processes facilitating–hampering at-homeness as described in our review requires further inquiry. We consider safety and connectedness to be more of a means of obtaining at-homeness and centeredness as the goal or foundation in its experience, although these aspects are clearly intertwined.

The essentiality of being centred is elaborated on by Relph (1976), who considers “home” to be “an irreplaceable centre of significance” (p. 39); that significant context where actions and intentions become signified with meaning and the recognized self. In this way, at-homeness will be closely connected with identity, spanning from the individual to close and distant places and locations, which are always experiential. Further, as Relph points out, places are experienced by the self as an insider or an outsider. Being centred in at-homeness means experiencing an existential insideness of belonging; being connected to self, others, things and so forth. Existential outsideness, on the other hand, “involves a self-conscious and reflective uninvolvement, an alienation from people and places, homelessness, a sense of the unreality of the world, and of not belonging” (Relph, 1976, p. 51), that is, being metaphorically homeless.

Our review clarifies that this unbelonging tends to be alienating and thus threatens the person's existence, which is elsewhere found to be associated with desolation and suffering (see, for example, Öhlén, 2004; Öhlén & Holm, 2006; Söderberg, Gilje, & Norberg, 1999). The belonging in at-homeness tends to be contemplative, allows for reflection and is associated with peace, consolation and permissive rest giving respite. For people with suicidal experiences, being connected is reported to also include a desire to be reachable (Talseth et al., 2003). From the perspective of healthcare providers, Söderberg et al. (1999) found facilitating “at-homeness” to be an essential aim for enrolled nurses confronted with ethically difficult situations in intensive care so that the suffering person could move beyond tragedy to fragility, paving the way for consolation and trustful responsibility.

According to Dyck et al. (2005), the insideness–outsideness belonging is a moral place, and as such is the setting where patients are nursed and reconstructed as a caregiving space. The values signified to such a space for giving care and for mediating a sense of being at-home need to be considered in order to facilitate at-homeness for people who are ill and lack wellness. In this process, Dyck et al. (2005) found the negotiation of bodies and homes as fields of knowledge to be of particular significance. In our view, these aspects of insideness and outsideness challenge other significant conceptualizations of wellness and thus need further elaboration.

General meanings of being safe, connected and centred as related to at-homeness have been elaborated in philosophy, which points to the significance for further philosophical inquiry of these empirically based aspects of at-homeness. Further, the relevance of using experiential and existential meanings of at-homeness underpins the potentiality for bridging the assumed gap between philosophical and everyday clinical discourse (Ahlzén, 2011) and indicating possibilities for clinical evaluations. In this way, being safe, connected and centred appear as essential aspects of at-homeness, representing aspects of operationalization of a different kind to that of Molony et al. (2007) measure for the experience of home consisting of “home,” “not home” and “boundary.” The results of this study could thus be a significant step in the process for further operationalization of “at-homeness.”

### Limitations

Specific limitations of this study need to be highlighted. The literature searches performed were probably incomplete since a systematic database search could not be carried out: studies related to “at-homeness” are not indexed with similar descriptive key terms. Even if we performed several manual searches including back and forth citation searches in addition to database searches, additional studies of interest might exist, probably in expanding fields such as geography of health and illness. Another limitation is the fact that some of the included studies emanate from one and the same project, and other studies from different projects were conducted by the same group of researchers. In this way, the discursive base for our analysis was more restricted than the studies which were included.

The study was limited to Scandinavian nursing research, which means inclusion of mutual influence among the studies reviewed. There might be a risk of delimiting the input and flow of ideas and “thinking,” especially in interpretative and inductive studies such as the ones reviewed. Nevertheless, the reviewed studies presenting aspects of at-homeness related to severe illness do not explicitly build upon the philosophical work of “homelikeness” (Svenaeus, 2000a, b), although we recognize a reception in the field of exhaustion disorder (Jingrot & Rosberg, 2008).

In all the reviewed studies, aspects of at-homeness were results in studies into other related phenomena. In most of the studies, interpretations most clearly revealed the aspects that build up the conceptualization put forward in our study. The empirical quotes that clearly illustrate “at-homeness” were few. Further, a significant feature of the included studies was that most were performed with a phenomenological hermeneutic analysis in which the results intertwined empirically based themes with ideas from existential philosophy. Another commonality for most is that they have been instrumental in Scandinavian interpretative methodological development (Lindseth & Norberg, 2004). Further, the reviewed studies emanate from a limited linguistic area and so the results might reflect aspects of discourses in Scandinavian nursing research and culture.

Although the reviewed studies included broad contexts of severe illness and receiving care in hospitals, home care, and hospice and palliative care, and these contexts were clearly related to all three aspects of at-homeness, caution should be exercised in the application of the findings. We suggest further research of at-homeness in diverse contexts.

A further remark is the ambiguity surrounding our analysis of themes from the reviewed studies and their discrimination into individual processes of the phenomenon. Readers will hardly be convinced that individual themes could not also be placed under other processes. However, we propose this not be regarded as a weakness; rather, it is in line with the existential and experiential character of at-homeness and the fact that the vast majority of the studies were inductively analysed out of participants’ experiences.

Finally, our analysis reveals a concept focusing on experiences of individuals at the micro level. The experience of being at-home as well as being metaphorically homeless is, of course, created contextually in an individual's sociopolitical spheres, as clearly demonstrated by the studies of Angus et al. (2005) and Dyck et al. (2005) informed by sociological theory and cultural geography. Thus, further elaboration on micro–macro links of at-homeness is merited, and the development of health and nursing geography appears to be especially promising in this regard (Giesbrecht, Crooks, & Stajduhar, 2014).

## Conclusions

In this review of interpretive studies related to severe and long-term illness conducted in Sweden, at-homeness despite illness and disease was conceptually found to be a significant process and state of wellness imbedded in the continuum of being metaphorically at-home and being metaphorically homeless. Being safe, being connected and being centred were discerned from the patients’ experiences to constitute essential aspects of at-homeness. These three empirically derived aspects of at-homeness indicate a need for further operationalization and instrument development. Note, however, that this has not resulted in our reaching full conceptual clarification, rather it is a further step in empirical investigation and theoretical development of “at-homeness,” inquiry into the possibility of hampering or facilitating at-homeness being a particular need here. Further development of at-homeness from a philosophical standpoint is also of particular significance. To conclude, we find this conceptualization to be of plausible significance for the evaluation of interventions aimed at enhancing wellness for people with severe long-term illness, such as the frail elderly, and people with chronic illness or palliative care needs.
